# The Impact of Surgeon Speciality Interest on Outcomes of Emergency Laparotomy in IBD

**DOI:** 10.1007/s00268-023-07051-z

**Published:** 2023-05-24

**Authors:** J. A. Bunce, B. Doleman, J. N. Lund, G. M. Tierney

**Affiliations:** 1 Division of Health Sciences and Graduate Entry Medicine, Faculty of Medicine, Royal Derby Hospital, University of Nottingham at Derby, Derby, UK; 2 Department of Colorectal Surgery, Royal Derby Hospital, Derby, UK

## Abstract

**Introduction:**

Emergency laparotomy may be required in patients with inflammatory bowel disease (IBD). NELA is the largest prospectively maintained database of adult emergency laparotomies in England and Wales and includes clinical urgency of the cases. The impact of surgeon subspeciality on outcomes after emergency laparotomy for IBD is unclear. We have investigated this association, according to the degree of urgency in IBD emergency laparotomy, including the effect of minimally invasive surgery (MIS).

**Methods:**

Adults with IBD in the NELA database between 2013 and 2016 were included. Surgeon subspeciality was colorectal or non-colorectal. Urgencies are ‘Immediate’, ‘2–6 h’, ‘6–18 h’ and ’18–24 h’. Logistic regression was used to investigate in-patient mortality and post-operative length of stay (LOS).

**Results:**

There was significantly reduced mortality and LOS in IBD patients who were operated on by a colorectal surgeon in the least urgent category of emergency laparotomies; Mortality adjusted OR 2.99 (CI 1.2–7.8) *P* = 0.025, LOS IRR 1.18 (CI 1.02–1.4) *P* = 0.025. This association was not seen in more urgent categories. Colorectal surgeons were more likely to use MIS, *P* < 0.001, and MIS was associated with decreased LOS in the least urgent cohort, *P* < 0.001, but not in the other urgencies.

**Conclusions:**

We found improved outcomes in the least urgent cohort of IBD emergency laparotomies when operated on by a colorectal surgeon in comparison to a non-colorectal general surgeon. In the most urgent cases, there was no benefit in the operation being performed by a colorectal surgeon. Further work on characterising IBD emergencies by urgency would be of value.

## Introduction

Emergency laparotomy may be required in patients with inflammatory bowel disease (IBD). Many of these operations are performed out of normal working hours by the on-call general surgeon. UK surgical training demands that all trainees obtaining Certification of Completion of Training (CCT) in general surgery are able to manage an unselected surgical take and perform the necessary operations via either an open or minimally invasive approach [1]. However, as a consultant, a general surgeon’s elective practice is often within a speciality interest area such as colorectal or hepatobiliary, whilst their emergency work remains general. IBD falls under the speciality interest of colorectal surgeons. This is supported by IBDUK standards which state that elective IBD operations should be performed by a colorectal surgeon [2]. There is evidence that mortality is significantly increased in emergency laparotomy when the pathology encountered is not in the speciality area (upper or lower gastrointestinal tract surgery) of the operating surgeon [3]. However, a recent cohort study of 913 patients suggests this may not be the case in IBD patients [4].

Inflammatory bowel disease may present acutely to surgeons in a number of ways. Some, with perforation or peritonitis will require immediate emergency surgery. Others will require expedited surgery through lack of response to maximal medical therapy [5]. Urgency of surgery is often classified using NCEPOD categories [6]. There is little data describing how outcomes for this cohort of patients differ according to urgency. The timing of surgery in IBD and the subspeciality interest of the operating surgeon are recognised as research priorities [7].

The National Emergency Laparotomy Audit (NELA) prospectively collects patient level data for emergency laparotomy in England and Wales with a case ascertainment of approximately 80%. The primary goal of the audit is to improve patient outcomes through identification of patient and organisational risk factors. Some key outputs include increasing consultant presence in theatre, post-operative critical care support, and for patients with a pre-operative risk of death of greater than 5%. Since it was first commissioned in 2013, NELA has observed a decrease in mortality and post-operative length of stay. Among the data collected are specimen histology, speciality interest of the operating surgeon and clinical urgency of the case, as defined by POSSUM and NCEPOD [8, 9].

The aim of this study is to investigate the impact of surgeon subspeciality interest, timing of surgery and surgical approach on outcomes for patients who have urgent or emergency surgery for IBD. It is thought that surgeon’s elective speciality interest may have an influence on IBD emergency outcomes through peri-operative decision making in these often-complex patients, as well as through differential experience with surgery of the lower GI tract.

## Methods

NELA, approved under Sect. 251 of the NHS Act 2006 by the Confidential Advisory Group, is commissioned to collect data on, understand and improve the care of patients undergoing emergency laparotomy in England and Wales; this process is outlined in the NELA annual reports[8]. For this analysis, data from December 2013 to December 2020 were extracted from the NELA dataset.

Patients were classified as having had an emergency laparotomy for inflammatory bowel disease if ulcerative colitis (UC) or Crohn’s disease (CD) was recorded in the histology domain of the NELA database. The primary procedure for that admission was the only one included in the analysis; planned or unplanned returns to theatre were not included to avoid increasing heterogeneity in the group. Complete data, including histology, was available between December 2013 and November 2016. After 2016, NELA no longer recorded histology and so data from 2017 to present has been excluded as the authors cannot be sure it accurately represents IBD. Patients with UC or CD were analysed grouped in a single cohort, here on referred to as ‘IBD’, and by specific IBD diagnosis.

NELA records the general surgery speciality interest of the consultant surgeon responsible for the case. For the purpose of this study, these were grouped as colorectal and non-colorectal.

The three cohorts; UC, CD and IBD (UC and CD combined) were analysed as groups divided by clinical urgency. NELA records clinical urgency categories as ‘18–24 h’, ‘6–18 h’, ‘2–6 h’, ‘ < 2 h’ and ‘immediate’ [8]. The last two categories have been grouped together as ‘ < 2 h’ due to the low numbers and the negative effect this would have on power. Outcome measures included in-hospital mortality and post-operative length of stay.

### Statistical analysis

Descriptive data are presented as median [interquartile range] and proportion (%) as appropriate. Logistic regression models were adjusted for a priori potential confounding variables. These included age, sex, ASA grade, cardiac and respiratory symptoms, ECG findings, systolic blood pressure, pulse rate, white cell count, creatinine, sodium, potassium, Glasgow coma score, high risk indication (perforation or ischaemia), anaemia (moderate or severe) and post-operative destination (ward versus higher level care) [8]. We could not add other a priori variables including frailty, CRP or lactate due to a large amount of missing data. Blood loss and soiling were not added as this may be related to surgical skill. Minimally invasive surgery (MIS) in IBD has been reported on in several cohort studies and is included in the NELA data [10]. Post hoc analysis of MIS has been included, defined as an operation that was completed laparoscopically, using logistic regression, to aid in discussion of the results.

Linear continuous predictors were identified using the lowess command of logit-transformed outcomes and the Box–Tidwell test. Nonlinear covariates were added as factor variables in known risk categories as per the NELA risk score publication [8]. This was to aid in the clinical interpretation of the results (as polynomials are difficult to interpret). To analyse binary outcomes, we used a Firth’s logistic regression model. This was chosen to overcome the model being compromised by perfect separation. Length of stay was analysed using negative binomial regression with clustered standard errors. Binary outcomes are reported as odds ratios (OR) and length of stay as incident rate ratios (IRR). Estimates are reported with 95% confidence intervals (CIs) and p values. All analyses were conducted using Stata Version V17.0.

## Results

We identified 3744 cases of emergency laparotomy for IBD between December 2013 and November 2016 in England and Wales. The patient characteristics by operating surgeon speciality interest are displayed in Table 1. When divided into cohorts according to clinical urgency, the largest group is those requiring surgery within 18–24 h, the lowest urgency. There is an upward trend in incidence of contamination and perforation as an intraoperative finding, as clinical urgency increases (Table 2).

**Table 1:** Characteristics of patients having emergency laparotomy according to IBD subtype and operating surgeon speciality interest. Values in parenthesis represent percentages unless otherwise stated

		UC		CD		IBD	
		Colorectal	Non-colorectal	colorectal	Non-colorectal	colorectal	Non-colorectal
Number (n)		909	271	1569	581	2478	852
Age (median [IQR])		43 (29–59)	51 (34–58)	39 (27–57)	42 (29–58)	40 (27–57)	44 (30–61)
Male		494 (54)	159 (59)	786 (50)	292 (50)	1280 (51.7)	451 (52.9)
ASA	1	42 (4.6)	13 (4.8)	132 (8.4)	55 (9.6)	174 (7.0)	68 (8.0)
	2	465 (51.2)	121 (44.6)	876 (55.8)	329 (57.7)	1341 (54.1)	450 (52.8)
	3	349 (38.4)	104 (38.4)	500 (31.9)	145 (25.4)	849 (34.3)	249 (29.2)
	4	50 (5.5)	32 (11.8)	55 (3.5)	40 (7)	105 (4.2)	72 (8.5)
	5	3 (0.3)	1 (0.4)	6 (0.4)	1 (0.2)	9 (0.4)	2 (0.2)
P-POSSUM predicted mortality (median [IQR])		3.9 (2–7.4)	5.3 (2.9–14.6)	3.1 (1.5–6.8)	3.3 (1.7–6.9)	3.2 (1.7–6.6)	3.8 (1.8–7.9
Urgency	< 2 h	88 (9.7)	34 (12.5)	125 (8.0)	67 (11.6)	213 (8.6)	101 (11.9)
	2–6 h	159 (17.5)	74 (27.3)	320 (20.4)	167 (28.9)	479 (19.3)	241 (28.3)
	6–18 h	240 (26.5)	79 (29.2)	514 (32.8)	192 (33.3)	754 (30.4)	271 (31.8)
	18–24 h	419 (46.2)	84 (31.0)	606 (38.7)	151 (26.2)	1025 (41.4)	235 (27.6)
Minimally invasive surgery		277 (30.5)	22 (8)	187 (11.9)	33 (5.7)	455 (19.4)	53 (6.6)

**Table 2:** The number of emergency laparotomies for each clinical urgency in UC, CD and IBD, with the associated incidence of intraoperative findings of peritoneal contamination and perforation. Values in parenthesis are percentages

		UC				CD				IBD			
		< 2 h	2–6 h	6–18 h	18–24 h	< 2 h	2–6 h	6–18 h	18–24 h	< 2 h	2–6 h	6–18 h	18–24 h
N		123	248	337	546	205	504	776	802	328	752	1113	1348
Contamination	Localised	18 (14.6)	44 (17.7)	28 (8.3)	30 (5.5)	43 (20.9)	147 (29.2)	187 (24.1)	169 (21.1)	61 (18.6)	191 (25.4)	215 (19.3)	199 (14.7)
	Generalised	23 (18.7)	54 (21.8)	17 (5.1)	15 (2.8)	64 (31.2)	137 (27.2)	64 (8.3)	22 (2.7)	87 (26.5)	191 (25.4)	81 (7.3)	37 (2.7)
Intraoperative finding of perforation		28 (22.8)	70 (28.2)	31 (9.2)	24 (4.4)	73 (35.6)	182 (36.1)	116 (14.9)	68 (8.5)	101 (30.8)	252 (33.5)	147 (13.2)	92 (6.8)

In general, in-patient mortality rate was higher when the patient was operated on by a non-colorectal surgeon in all urgency cohorts except 6–18 h. After adjusting for confounding factors, this reached statistical significance for the IBD cohort in the least urgent category of 18–24 h with a mortality rate for colorectal surgeons of 1.4% and non-colorectal surgeons of 4.8% (adjusted OR 2.9, 95% CI 1.2 to 7.8, *P* = 0.025). This is also true of the least urgent CD cases where the mortality for colorectal surgeons was 0.9% and non-colorectal surgeons was 4.1% (adjusted OR 5.39, 95% CI 1.3–21.7, *P* = 0.017). This increased mortality is not seen in more urgent cases of IBD, UC or CD cohorts (Tables 3, 4, 5). However, imprecision was observed in the UC mortality analysis is due to low numbers.

**Table 3:** Firth logistic regression and negative binomial regression showing the difference in outcome between colorectal and non-colorectal surgeon for each urgency group in patients with IBD. Values in parenthesis are 95% confidence intervals

	IBD															
	< 2 h				2–6 h				6–18 h				18–24 h			
	Colorectal	Non-colorectal	Adjusted OR/IRR	*P*	Colorectal	Non-colorectal	Adjusted OR/IRR	*P*	Colorectal	Non-colorectal	Adjusted OR/IRR	*P*	Colorectal	Non-colorectal	Adjusted OR/IRR	*P*
In-hospital mortality (%)	8.2	9.5	1.2 (0.3–4.9)	0.83	5.9	10.1	1.2 (0.5–2.9)	0.59	2.6	1.5	0.6 (0.2–1.9)	0.38	1.4	4.8	2.9 (1.2–7.8)	0.025
Median post op length of stay (Days)	11.1	13.6	0.9 (0.8–1.3)	0.81	11.1	12.4	0.9 (0.8–1.1)	0.31	9.1	10.1	0.99 (0.9–1.1)	0.79	8.9	10.1	1.2 (1.0–1.4)	0.025

**Table 4:** Firth logistic regression and negative binomial regression showing the difference in outcome between colorectal and non-colorectal surgeon for each urgency group in patients with CD. Values in parenthesis are 95% confidence intervals

	CD															
	< 2 h				2–6 h				6–18 h				18–24 h			
	Colorectal	Non-colorectal	Adjusted OR/IRR	*P*	Colorectal	Non-colorectal	Adjusted OR/IRR	*P*	Colorectal	Non-colorectal	Adjusted OR/IRR	P	Colorectal	Non-colorectal	Adjusted OR/IRR	P
In-hospital mortality (%)	8.8	6.2	0.27 (0.02–2.5)	0.26	3.9	6.1	1.18 (0.2–5.6)	0.83	2.7	1.6	1.33 (0.3–4.5)	0.65	0.9	4.1	5.39 (1.3–21.7)	0.017
Median post op length of stay (Days)	10.9	11.7	0.99 (0.7–1.3)	0.91	10.7	10.1	0.97 (0.8–1.1)	0.73	8.9	9.1	0.92 (0.8–1)	0.11	8.6	8.9	1.04 (0.8–1.3)	0.73

**Table 5:** Firth logistic regression and negative binomial regression showing the difference in outcome between colorectal and non-colorectal surgeon for each urgency group in patients with UC. Values in parenthesis are 95% confidence intervals

	UC															
	< 2 h				2–6 h				6–18 h				18–24 h			
	Colorectal	Non-colorectal	Adjusted OR/IRR	P	Colorectal	Non-colorectal	Adjusted OR/IRR	P	Colorectal	Non-colorectal	Adjusted OR/IRR	P	Colorectal	Non-colorectal	Adjusted OR/IRR	P
In-hospital mortality (%)	7.4	16.7	1.79 (0.2–14.3)	0.58	9.9	18.9	0.89 (0.3–2.8)	0.84	2.6	1.3	0.89 (0.1–13.6)	0.93	2.2	6.1	2.37 (0.4–15)	0.36
Median post op length of stay (Days)	11.8	17.8	1.21(0.8–1.7)	0.24	12.9	16.8	0.85 (0.7–1)	0.11	9.9	14.1	1.1 (0.9–1.4)	0.19	9.8	13.1	1.4 (1.2–1.6)	< 0.001

There was an upward trend in post-operative length of stay in patients operated on by non-colorectal surgeons in comparison to colorectal surgeons in almost all cohorts. In IBD patients, this reached statistical significance in the least urgent cohort with a median post-operative length of stay for colorectal surgeons of 8.9 days in comparison to 10.1 days for non-colorectal surgeons (adjusted IRR 1.2, 95% CI 1.02 to 1.36, *P* = 0.025). This difference is also seen in the least urgent UC cohort where the post-operative length of stay increases from 9.8 days for colorectal surgeons to 13.1 days for non-colorectal surgeons (adjusted IRR 1.4 95% CI 1.2–1.6, *P* < 0.001). We observed no difference in post-operative length of stay for the CD cohorts between colorectal and non-colorectal surgeons.

After adjusting for urgency of operation in regression analysis, more patients operated on by a colorectal surgeon underwent MIS, in comparison to those operated on by a non-colorectal surgeon, in IBD 19.4% MIS when operated on by a colorectal surgeon in comparison to 6.6% when operated on by a non-colorectal general surgeon (adjusted OR 3.06, 95% CI 2.3 to 4.1, *P* < 0.001), in UC 31.2% MIS when operated on by a colorectal surgeon in comparison to 8.5% when operated on by a non-colorectal general surgeon (adjusted OR 4.6, 95% CI 2.9 to 7.4, *P* < 0.001) and in CD 12.3% MIS when operated on by a colorectal surgeon in comparison to 5.6% when operated on by a non-colorectal general surgeon (adjusted OR 2.03, 95% CI 1.4 to 2.9, *P* < 0.001). When adjusting for confounding variables, there was no association between MIS and mortality. There was however a significant association between MIS and post-operative length of stay (LOS) in the least urgent IBD, UC and CD cases; For IBD, LOS after MIS was 7 days in comparison to LOS after open surgery of 9.8 days ( adjusted IRR 1.3, 95% CI 1.1–1.4, *P* < 0.001). In UC, LOS after MIS was 7.9 days in comparison to LOS after open surgery of 10.9 days (adjusted IRR 1.3, 95% CI 1.1–1.5, *P* < 0.001). In CD, LOS after MIS was 6.1 days in comparison to LOS after open surgery of 8.9 days (adjusted IRR 1.2, 95% CI 1.04–1.5, *P* = 0.02). This association between MIS and post-operative LOS was not seen in the more urgent cases (Tables 6, 7, 8).

**Table 6:** Negative binomial regression showing the difference in median post-operative length of stay between MIS and open surgery in each urgency group of patients with IBD. Values in parenthesis are 95% confidence intervals

	IBD															
	< 2 h				2–6 h				6–18 h				18–24 h			
	MIS	Open	Adjusted OR/IRR	P	MIS	Open	Adjusted OR/IRR	P	MIS	Open	Adjusted OR/IRR	P	MIS	Open	Adjusted OR/IRR	P
Median post op length of stay (Days)	9.9	12.0	0.7 (0.5–1.1)	0.09	8.0	11.8	1.1 (0.9–1.4)	0.31	7.9	9.9	1.1 (0.9–1.2)	0.26	7	9.8	1.3 (1.1–1.4)	< 0.001

**Table 7:** Negative binomial regression showing the difference in median post-operative length of stay between MIS and open surgery in each urgency group of patients with UC. Values in parenthesis are 95% confidence intervals

	CD															
	< 2 h				2–6 h				6–18 h				18–24 h			
	MIS	Open	Adjusted OR/IRR	*P*	MIS	Open	Adjusted OR/IRR	*P*	MIS	Open	Adjusted OR/IRR	*P*	MIS	Open	Adjusted OR/IRR	*P*
Median post op length of stay (Days)	9.9	11.6	0.8 (0.5–1.2)	0.33	9.9	10.6	0.9 (0.6–1.3)	0.63	7.1	9.1	1.1 (0.9–1.3)	0.62	6.1	8.9	1.2 (1.04–1.5)	0.02

**Table 8:** Negative binomial regression showing the difference in median post-operative length of stay between MIS and open surgery in each urgency group of patients with CD. Values in parenthesis are 95% confidence intervals

	UC															
	< 2 h				2–6 h				6–18 h				18–24 h			
	MIS	Open	Adjusted OR/IRR	*P*	MIS	Open	Adjusted OR/IRR	*P*	MIS	Open	Adjusted OR/IRR	*P*	MIS	Open	Adjusted OR/IRR	*P*
Median post op length of stay (Days)	9.4	15.1	0.7 (0.5–1.2)	0.20	7.5	15.7	1.4 (1.1–1.9)	0.007	8.9	11.8	1.2 (0.9–1.4)	0.08	7.9	10.9	1.3 (1.1–1.5)	< 0.001

## Discussion

This study builds on previous work done in this important area of emergency surgery. Boyd-Carson et al. found that an emergency laparotomy performed by a surgeon whose elective special interest is not in the area of the pathology, is associated with significantly increased mortality [3]. This supported the work done by Biondo et al. in colorectal patients [11]. However, in 2021, Macfarlane et al. found no significant difference in outcome in IBD patients operated on by colorectal surgeons in comparison to non-colorectal surgeons [4]. That study reported on a broad range of pre-operative factors using hospital episode statistics data which they acknowledge as being limited in a number of ways. This is the first analysis of emergency laparotomy for all patients with inflammatory bowel disease taken from NELA, the largest prospectively maintained database of laparotomy patients in England and Wales. As a result of this large dataset, we are able to build on the previous work in this area with a higher level of data granularity. This allowed us to perform subgroup analysis on CD and UC as separate cohorts and look at each clinical urgency independently.

When sub-dividing cases by clinical urgency, we can see the majority fall into the least urgent category. In the published literature, this 18 to 24-h urgency is a grey area, often classified as urgent, or expedited. Our data show that this cohort has a relatively low incidence of perforation and peritoneal contamination in comparison to the other urgency categories. These patients may have had a trial of medical management before proceeding to surgery and could be considered ‘semi-elective’. They may have been discussed in the IBD multi-disciplinary team meeting (MDT) and had more extensive pre-operative optimisation. In this group, our data show a significant difference in outcome for patients operated on by a colorectal surgeon in comparison to a non-colorectal surgeon for both mortality and post-operative length of stay.

It is possible that this reflects peri-operative decision making; colorectal surgeons are more likely to be part of the IBD MDT, have closer working relationships with luminal gastroenterology colleagues and more familiar with the pre- and post-operative management of IBD. Another consideration is the role of MIS. Multiple cohort studies have shown benefit in IBD emergencies, and our data agrees that there is a benefit to length of stay [10]. In our data, this benefit is most significant in the least urgent cohort. We have also demonstrated that colorectal surgeons are significantly more likely to use MIS in IBD emergencies. It may be that the LACES trial will inform further on this [12].

Another significant finding is the data showed no difference in outcome according to the operating surgeon in more urgent IBD emergencies, that is surgery required within 18 h. Table 3 demonstrates the increase in incidence of perforation and contamination in these more urgent IBD cases. A previous study has demonstrated a 6% increase in risk of death per hour delay in source control after a perforated peptic ulcer [13]. Other cohort studies have demonstrated a similar relationship between delays to theatre and outcome in IBD patients [14–17]. The likely reason for our observed lack of surgeon specific effect on outcomes in more urgent patients is because the priority in sepsis is source control rather than IBD specific management. This aligns with the 2021 Joint Colleges of Surgical Training (JCST) curriculum requirement that an emergency general surgeon must be able to perform the required surgical management of an acute abdomen [1].

Current guidelines for elective surgery in inflammatory bowel disease recommend a colorectal surgeon with an interest in IBD and use of MIS whenever possible [2]. There are, as yet no such guidelines for emergency procedures in this patient group. Analysis of this NELA data suggests that similar recommendations to those that apply to elective patients, could apply to patients in the low urgency emergency setting. The findings from this analysis that a large number of IBD patients are ‘non urgent’ emergencies may also indicate a lack of elective colorectal operating lists available for IBD. Recommendations for increased availability of colorectal surgeons on call or elective operating lists might be impractical for some trusts with limited numbers of surgeons providing emergency care.

To capture clinical urgency, we have used the target time for the patient arrive in theatre which is reflected in the booking urgency. The recommendation by NCEPOD is that the consultant in charge of the care of that patient should decide the booking urgency, and it should reflect their clinical condition, we accept that this may be subject to bias [6]. Actual measured time taken to arrive in theatre could also be used however is affected by organisational limitations so may not accurately reflect the patient’s clinical condition.

### Limitations

NELA case ascertainment is estimated at 80%, so this dataset is incomplete. Nonlinear predictors were added as factor variables using NELA defined cut offs for low, normal and high. This was chosen over transformed polynomials as it has easier clinical interpretation but can result in loss of information. This is a retrospective observational study, reporting associations. A randomised control trial would be required to report causation. We were also unable to add other, potentially important confounders such as frailty and lactate because of missing data. As mentioned in methods, NELA has only recorded histology data from 2013 to 2016; however, this still represents one of the largest cohort studies for IBD in the UK and a valuable resource.

## Conclusion

Emergency laparotomy for IBD where there is less clinical urgency is associated with decreased mortality and length of stay when performed by a colorectal surgeon in comparison to a non-colorectal general surgeon. Where there is greater clinical urgency, there is little benefit of MIS or a colorectal surgeon above a non-colorectal general surgeon. This may have significant implications for how emergency IBD cases are classified and how emergency general surgery departments are structured. Further work in the area of urgency and timing of emergency surgery may help further distinguish these two groups of patients from one another.
